# Temporal patterns of thyroid gland maturation and skin metamorphosis in terrestrial indirect-developing de Villiers moss frogs (*Arthroleptella villiersi* Hewitt, 1935)

**DOI:** 10.1186/s12983-026-00631-4

**Published:** 2026-09-23

**Authors:** Benjamin Naumann, Susan Schweiger, Hendrik Müller

**Affiliations:** 1 Institut für Biowissenschaften, Allgemeine und Spezielle Zoologie, Universität Rostock, Rostock, Germany; 2 Institut für Zoologie und Evolutionsforschung, Friedrich-Schiller-Universität, Jena, Germany; 3 Zoologische Sammlung, Zentralmagazin Naturwissenschaftlicher Sammlungen, Martin-Luther-Universität Halle-Wittenberg, Halle (Saale), Germany

**Keywords:** Evolution, Epidermis, Heterochrony, Terrestrialization, Tadpole

## Abstract

**Background:**

Anurans show diverse life histories ranging from the ancestral aquatic indirect development to derived terrestrial developmental modes that often correlate with the reduction of larval characters. The de Villiers moss frog *Arthroleptella villiersi* Hewitt, 1935 (Pyxicephalidae) exhibits terrestrial indirect development, hatching as endotrophic tadpoles that undergo metamorphosis within the jelly nest. We investigated the timing and histology of skin and thyroid gland development of *A. villiersi* to test whether metamorphic rearrangements were conserved and to assess the role of heterochrony in terrestrialization of anuran life histories.

**Results:**

Histological analyses revealed a conserved sequence of thyroid maturation compared to aquatic-indirect and terrestrial direct developing anurans. Skin development recapitulates tadpole typical features, including unicellular glands, a two-layered epidermis, and neuromasts. Subsequent metamorphic rearrangements lead to a three-layered epidermis, stratum corneum, multicellular mucus and granular glands, and reorganized melanophore layers. Neuromasts appear at hatching, differentiate, and degenerate shortly before metamorphosis, correlating with thyroid maturation. Heterochrony plots revealed minor shifts in timing of skin development, including pre-displacement of the development of dermal melanophores and the post-displacement of multicellular granular glands, compared to aquatic indirect-developing anurans.

**Conclusions:**

Our findings demonstrate that *A. villiersi* retains a conserved metamorphic remodeling of the skin that correlates with thyroid maturation. The observed minor heterochronic shifts highlight the modularity of skin development without evidence for temporal co-dissociations and therefore indicate an integrated “skin unit of evolution”. This indicates that evolutionary transitions to terrestrial life histories in anurans can occur without a fundamental reorganization of skin developmental pathways.

**Supplementary Information:**

The online version contains supplementary material available at https://doi.org/10.1186/s12983-026-00631-4.

## Background

Most animals (Metazoa) develop indirectly, with a morphologically and ecologically distinct larval stage that undergoes metamorphosis into the adult form [1]. The often profound metamorphic rearrangement of the body potentially decouples larval and adult morphology and is thought to enable the independent adaptation to differing selective pressures [2, 3]. In several of these ancestrally indirect-developing lineages, the larval phase has been secondarily reduced or lost, leading to a more gradual development of the adult morphology [1]. With the reduction or loss of the larval stage within the life history and the connected relaxation of selective pressures on larval-specific traits, the following question arises: to what extant are larval characters conserved or lost in taxa that show a reduced larval phase? Answering this question will help to identify units of evolution as well developmental constraints that may have influenced the evolution of direct development different animal groups [4].

**Fig. 1:**
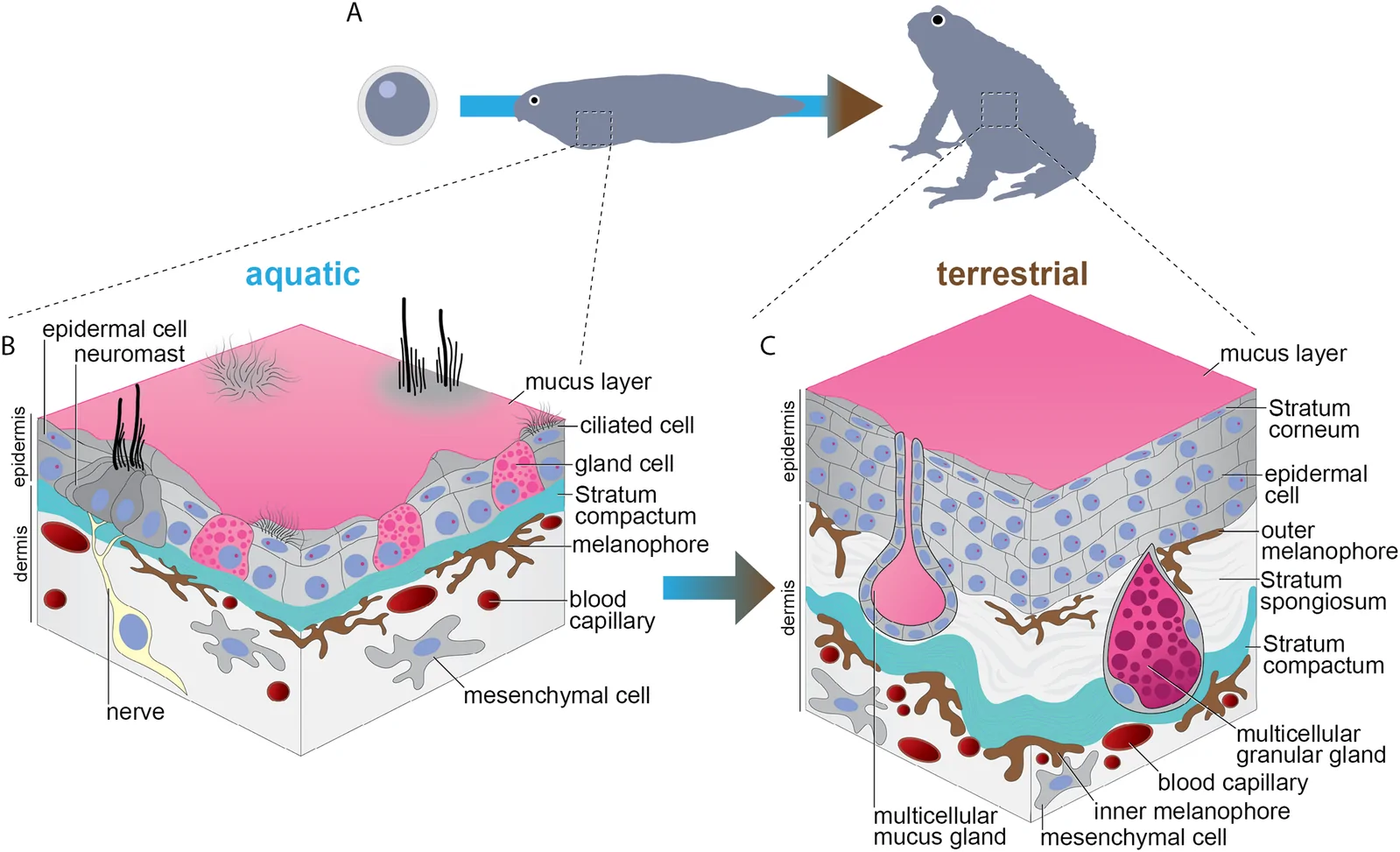
A, Schematic illustrations of the ancestral aquatic indirect life history in Anura. B, Schematic illustration of the skin of a free-living, aquatic tadpole. C, Schematic illustration of the skin of a postmetamorphic anuran

Extant amphibians (Lissamphibia), comprising caecilians (Gymnophiona), salamanders (Caudata) and frogs (Anura), are well-suited to addressing this question. Their ancestral life history is characterized by aquatic-indirect development, involving an aquatic larva that transforms via a thyroid hormone-dependent metamorphosis into a semi-terrestrial adult [5, 6]. All three amphibian groups have evolved diverse life histories, including different aquatic, semi-terrestrial, and fully terrestrial reproductive modes, with varying degrees of larval reduction up to its complete loss in terrestrial direct-developing taxa [7]. With over 8000 described species (amphibiaweb.org, 03.08.2026), anurans are the evolutionarily most successful amphibian group in terms of species richness. This evolutionary success is largely attributed to their diverse indirect life histories, which include highly specialized free-swimming feeding larvae known as tadpoles [8]. Within anurans, terrestrial reproductive modes, including the reduction (terrestrial-indirect development) or even complete loss of the free-living tadpole phase (direct development) (Fig. 1A), have evolved independently more than 20 times [9].

Studies on terrestrial direct-developing anurans of the genera *Eleutherodactylus* and *Arthroleptis* have revealed evolutionary losses of many tadpole-typical characters such as the rostral jaw cartilages, adhesive glands, lateral line organs, and extensive midgut coiling [10–12]. Other tadpole-typical characters such as a long tail, an epidermal covering of gills and forelimbs (operculum) and a two-layered epidermis with unicellular glands still develop but are remodelled early in ontogeny [11–14]. Alongside characters that start to develop in a mid-metamorphic state, these patterns support the hypothesis that the evolution of anuran direct development involved large-scale heterochronic shifts [15]. Pre-displacement of pituitary and thyroid gland maturation within the brain-pituitary-thyroid-axis (BPT-axis) were proposed as the main driver of heterochronic shifts of other organs and structures (11, 16, 17). A dependence on thyroid hormone (TH) has been shown for some developmental aspects in the direct-developer *Eleutherodactylus coqui* [16]. But is heterochrony an equally important pattern underlying the evolution of terrestrial indirect developing life histories of amphibians?

Terrestrial indirect-developing anurans have non-feeding tadpoles that hatch from terrestrial eggs. These tadpoles complete metamorphosis within a terrestrial foam or jelly nest, which represents a radically different habitat compared to that of a feeding aquatic tadpole [8]. Testing the importance of heterochrony in shaping the diversity of anuran life histories and the underlying role of thyroid gland maturation requires detailed comparative data from anurans with diverse reproductive and developmental modes. Such data, including the developmental timing of various organ systems, can be analysed using formalized techniques such as heterochrony plots to identify patterns of temporal shifts and covariation. These patterns may help define developmental modules and propose hypotheses about the units of evolution of species for which functional experiments, such as inhibitor assays or gene knock-downs, are currently not possible [4].

The present study investigates the timing of skin and thyroid gland development in the terrestrial indirect-developing anuran species *Arthroleptella villiersi* Hewitt, 1935 (Pyxicephalidae). In *A. villiersi*, eggs are laid on land beneath vegetation, in loose soil or amongst organic matter, and are surrounded by a moist jelly mass. Non-feeding tadpoles relying on their yolk reserves (endotrophic) hatch from these eggs at an early metamorphic stage, and continue their development within the jelly nest until metamorphosis is completed and they emerge as small froglets [17]. This alternative life history of endotrophic anurans combines characters of both terrestrial-direct development and aquatic-indirect development, including an apparent metamorphosis [17, 18]. While many organs are subject to rearrangement during anuran metamorphosis, the skin undergoes one of the most extensive changes and has been suggested as a model system for investigating the evolutionary alteration of ontogeny in anurans [11, 19]. The skin of pre-metamorphic tadpoles is adapted to an aquatic habitat and consists of a two-layered epidermis that includes unicellular glands that produce an outer mucus layer. Neuromasts, organized in distinct lateral lines, are embedded within this larval epidermis. The underlying dermis is a mesenchyme that includes a thin stratum compactum and melanophores (Fig. 1B). During pro-metamorphosis, most epidermal cells degenerate except those connected to the basal lamina. These cells then begin to proliferate again during the metamorphic climax. In most anurans investigated, neuromasts degenerate alongside the larval epidermis, persisting only during the post-metamorphic phase in taxa with aquatic adults, such as *Xenopus* [20]. The post-metamorphic epidermis mostly forms a five- to seven-layered epithelium with an outer keratinized stratum corneum and various types of multicellular glands. The dermis contains a stratum spongiosum and a thick stratum compactum surrounded by an outer and an inner melanophore layer (Fig. 1 (C)) [11, 21, 22]. Morgan et al. [18] described a conserved gross pattern of skin metamorphic rearrangement in *Arthroleptella lightfooti* (Boulenger, 1910) when compared to *Xenopus laevis* Daudin, 1802. However, information on the exact timing of specific developmental events, such as the emergence of placodes or the maturation and degeneration of neuromasts, is lacking. This lack hampers detailed comparisons necessary for identifying potential heterochronic shifts. To address this we [1] investigated the skin development and metamorphic rearrangement in *A. villiersi* in detail to determine if the developmental pattern present in aquatic indirect developing species is conserved and [2] compared the results to anurans with different reproductive modes in order to identify the pattern of heterochronic shifts in skin development, which will help to identify potential units of evolution and establish hypotheses on potential developmental constraints channelling their evolutionary trajectory.

## Methods

### Specimens

Embryos, hatchlings and adults of *A. villiersi* were collected in different locations of the Western Cape, South Africa (CapeNature collection permit numbers: 0035-AAA004-01041, 0056-AAA041-00073). Clutches were transferred to Petri dishes and maintained under field conditions and embryos were sampled at regular intervals (every 1–2 days). Eggs were incubated at maximum daytime temperatures of approximately 17 °C and nighttime temperatures of approximately 8.5 °C; mean temperatures were approximately 13 °C during the day and 10 °C at night. Moisture and parental condition were recorded. Specimens were euthanized using tricaine methanesulfonate (MS222; Fluka), fixed in 4% buffered formalin, and subsequently stored in 70% ethanol. Conspecificity of egg clutches was confirmed by raising individual eggs until hatching and by the species status of the egg-guarding male [23]. For each developmental stage examined herein, one individual was examined externally to describe the pigmentation pattern and another individual was sectioned to follow the histological changes of the skin. For further details on specimen collection and staging see Schweiger et al. [24]. Histological sections were examined for single individuals at stages 5, 6, 8–9, 10, 12, 13, 15, 17, 19, 21, and 22, and whole mounts for single individuals at stages 5, 7, 8, 9, 10, 12, 15, 17, 19, 21, and 22.

### Photographs and Image processing

Photographs of whole specimens were taken using a Zeiss Discovery V12 stereomicroscope equipped with a Zeiss AxioCam. Brightness and contrast were adjusted, and background noise was removed using Adobe Photoshop CS6.

### Histological sectioning and staining

Embryos were prepared for sectioning as described previously [11]. Specimens at developmental stages 4 and 6/7 were embedded in Technovit (Kulzer R0010022), sectioned at 3 μm and stained with a mixture of basophilic Methylene blue and acidic Fuchsin [25]. Embryos from stage 8 on were decalcified for up to three days (Osteomoll, Merck), embedded in Paraplast (Roth, X881.1), sectioned at 7 μm and stained either with Heidenhain’s Azan or Goldner’s Trichrom [25]. All sections were prepared with a Microm HM 360 (Zeiss). Photographs were taken using a Leica DM750.

### Histological measurements

We measured follicle volume and follicle cell height and combined these with histological descriptions to estimate thyroid gland activity [26, 27]. As a third indicator of thyroid gland activity, the total follicle volume of each specimen analysed was plotted on a logarithmic scale. Measurements and 3-dimensional reconstructions of single follicle cell volumes were obtained from digitized and aligned serial sections using Amira 5.4.2 (FEI Visualization, Science Group, Bordeaux, France). Follicle cell heights were measured from digitized sections using ImageJ [28]. Shapiro-Wilk tests (*n* < 50) or Shapiro-Francia tests (*n* > 50) were performed using the GIGAcalculator [29] to test for a normal distribution of measurements. All follicle cell height measurements showed normal distributions (Shaprio-Francia test p-value = 0), except for stage 10 (Shapiro-Wilk p-value = 0.157). All single follicle volumes showed a normal distribution (Shapiro-Wilk test or Shapiro-Francia test p-value = 0), except for stage 10, where the n (6 follicles) was too low to perform a test. Two-sample T-Tests and diagrams were prepared using Microsoft Excel 2021. All measured values and p-values are given in Supplementary Material 1.

### Heterochrony plots

A heterochrony plots represents a correlation plot in which the timing of developmental events in one taxon (x-axis) is plotted against the timing of homologous events in another taxon (y-axis) [4]. If the developmental staging tables of both taxa are comparable, homologous events showing a similar timing (an exact correlation) come to lie on a line with an inclination of approximately 45 degrees. If the timing is different, the resulting point in the plot will deviate from this 45-degrees reference line indicating a heterochronic shift. For a more detailed explanation and discussion of heterochrony plots see Schlosser [4]. In this study, 47 developmental events (13 developmental events of the skin and 35 general events) were conceptualized (Table 1). Causally linked events were grouped into nine developmental suites indicated by Roman numerals. Events were excluded from a plot when data on the developmental timing were not available for both taxa. Linear regression lines are given for the suites to roughly estimate their developmental rates. To facilitate a more comprehensive comparison of taxa with different staging tables (e.g., Gosner [33] with 46 stages vs. Townsend and Stewart [12] with 15 stages), five comparable ontogenetic phases were defined based on very general developmental events and indicated by dotted lines in each plot. Phase a (“pre-pharyngula” phase): fertilization until the last stage before the emergence of optic and gill arch bulges. Phase b (“pharyngula and post-pharyngula” phase): appearance of optic and gill arch bulges as well as the elongation of the tail bud until the last stage before the visibility of the pupil, the darkening of the iris as well as the maximum extension of the operculum (additionally the presence of forelimb buds beneath the operculum and a paddle-shaped foot in direct-developing species). Phase c (“tadpole” phase): first appearance of the of the pupil, the darkening of the iris as well as the maximum extension of the operculum (additionally the presence of forelimb buds beneath the operculum and a paddle-shaped foot in direct-developing species) until the last stage before first signs of tail regression. Phase d (“metamorphosis” phase): onset of tail regression until one stage before the completion of metamorphosis defined by the complete disappearance of a tail stub. Phase e (“postmetamorphosis” phase): complete absence of a tail stub onwards (see Table 2 for specific stages). To avoid overestimating minor temporal differences between taxa that may be artefacts based on different levels of detail in staging tables (see [4] for more information on this topic), only events occurring in different ontogenetic phases were scored as exhibiting a heterochronic shift. For example, Phase B in the taxon on the x-axis versus Phase C in the taxon on the y-axis. A difference within an ontogenetic phase also represents a sequence heterochronic shift (at least with the events that define the phase, but often with more events) or a telescoping dissociation *sensu* Schlosser [4].

When used in a phylogenetic context, heterochrony plots provide information about the direction of heterochronic shifts compared to an ancestral pattern [4]. Data from the aquatic indirect-developing species *Xenopus laevis* [30, 31] and *Rhinella arenarum* [32, 33] were therefore plotted against each other to determine the similarity in the timing of developmental events and to test their potential for exemplifying ancestral patterns in comparison with anurans exhibiting more derived developmental modes (Fig. 7A). The resulting heterochrony plot shows comparable developmental timing in both species, as indicated by the similar inclinations of regression lines and the close clustering of the developmental suites. Due to the comparable timing and the more detailed data on skin developmental events, *R. arenarum*, staged using the Gosner staging-system [33], was selected to represent the plesiomorphic condition in subsequent plots.

**Table 1:** Developmental events investigated in the heterochrony plots

event/suite number	developmental events	*Arthroleptella villiersi*		*Arthroleptis wahlbergii*		*Rhinella arenarum*		*Xenopus laevis*	
I	skin suite	stages of Schweiger et al., 2026	references	TS-stages (Schweiger et al., 2017)	references	Gosner-stages	references	NF-stage	reference
1	stratum compactum starts to develop	10	this study	8	Naumann et al., 2021	not available	-	50	Seki et al., 1989
2	stratum compactum shows increased thickness	12	this study	11	Naumann et al., 2021	30	Regueira et al., 2015	56	Seki et al., 1989
3	intracellular yolk granules absent	10	this study	10	Naumann et al., 2021	not available	-	-	-
4	dermal melanophores present	10	this study	6	Naumann et al., 2021	41	Regueira et al., 2015	-	
I.I	unicellular gland cell suite								
5	unicellular mucus glands present	10	this study	6	Naumann et al., 2021	30	Regueira et al., 2015	-	-
6	unicellualr mucus glands absent	12	this study	11	Naumann et al., 2021	39	Regueira et al., 2015	-	-
I.II	multicellular gland cell suite								
7	stratum spongiosum present	12	this study	11	Naumann et al., 2021	42	Regueira et al., 2015	57	Seki et al., 1989
8	multicellular gland anlagen	12	this study	11	Naumann et al., 2021	36	Regueira et al., 2015	57	Seki et al., 1989
9	multicellular granular glands present	17	this study	12	Naumann et al., 2021	39	Regueira et al., 2015	60	Seki et al., 1989
10	multicellular mucus glands present	13	this study	12	Naumann et al., 2021	41	Regueira et al., 2015	60	Seki et al., 1989
I.III	epidermal cell layer suite								
11	epidermis has up to two layers	8	this study	5	Naumann et al., 2021	30	Regueira et al., 2015	-	-
12	epidermis has three or more layers	19	this study	12	Naumann et al., 2021	43	Regueira et al., 2015	51	Seki et al., 1989
13	stratum corneum present	19	this study	13	Naumann et al., 2021	not available	-	58	Seki et al., 1989
II	hind limb suite								
14	fist anlage of the hind limb	4	Schweiger et al., 2026.; Morgan et al., 1989	4.5	Schweiger et al., 2017	26	Gosner, 1960; Regueira et al., 2015	48	Nieuwkoop and Faber, 1994
15	starting elongation of the hind limb buds	7	Schweiger et al., 2026.; Morgan et al., 1989	6	Schweiger et al., 2017	27	Gosner, 1960; Regueira et al., 2015	49	Nieuwkoop and Faber, 1994
16	limb paddle present	8	Schweiger et al., 2026.; Morgan et al., 1989	7	Schweiger et al., 2017	31	Gosner, 1960; Regueira et al., 2015	53	Nieuwkoop and Faber, 1994
17	toe 3-5 distinct	9	Schweiger et al., 2026.; Morgan et al., 1989	8	Schweiger et al., 2017	36	Gosner, 1960; Regueira et al., 2015	54	Nieuwkoop and Faber, 1994
18	toe 1-5 present	10	Schweiger et al., 2026.; Morgan et al., 1989	10	Schweiger et al., 2017	37	Gosner, 1960; Regueira et al., 2015	56	Nieuwkoop and Faber, 1994
III	forelimb suite								
19	first anlage of the forelimb buds	7	Schweiger et al., 2026.; Morgan et al., 1989	4.5	Schweiger et al., 2017	not available	Gosner, 1960; Regueira et al., 2015	50	Nieuwkoop and Faber, 1994
20	starting elongation of the forelimb buds	8	Schweiger et al., 2026.; Morgan et al., 1989	6	Schweiger et al., 2017	not available	Gosner, 1960; Regueira et al., 2015	51	Nieuwkoop and Faber, 1994
21	all 4 fingers differentiated	11	Schweiger et al., 2026.; Morgan et al., 1989	10	Schweiger et al., 2017	not available	Gosner, 1960; Regueira et al., 2015	56	Nieuwkoop and Faber, 1994
IV	tail suite								
22	tail bud present	4	Schweiger et al., 2026.; Morgan et al., 1989	4.0	Schweiger et al., 2017	17	Gosner, 1960; Regueira et al., 2015	26	Nieuwkoop and Faber, 1994
23	starting elongation of the tail bud	5	Schweiger et al., 2026.; Morgan et al., 1989	4.3	Schweiger et al., 2017	18	Gosner, 1960; Regueira et al., 2015	28	Nieuwkoop and Faber, 1994
24	fin folds present	8	Schweiger et al., 2026.; Morgan et al., 1989	5	Schweiger et al., 2017	20	Gosner, 1960; Regueira et al., 2015	28	Nieuwkoop and Faber, 1994
25	vascularized translucent fin folds	9	Schweiger et al., 2026.; Morgan et al., 1989	7	Schweiger et al., 2017	22	Gosner, 1960; Regueira et al., 2015	43	Nieuwkoop and Faber, 1994
26	tail begins to regress	12	Schweiger et al., 2026.; Morgan et al., 1989	12	Schweiger et al., 2017	40	Gosner, 1960; Regueira et al., 2015	58	Nieuwkoop and Faber, 1994
27	tail regressed to small stump	21	Schweiger et al., 2026.; Morgan et al., 1989	15	Schweiger et al., 2017	45	Gosner, 1960; Regueira et al., 2015	65	Nieuwkoop and Faber, 1994
28	tail resorbed	22	Schweiger et al., 2026.; Morgan et al., 1989	16	Schweiger et al., 2017	46	Gosner, 1960; Regueira et al., 2015	66	Nieuwkoop and Faber, 1994
V	opercular fold suite								
29	opercular fold first evident	7	Schweiger et al., 2026.; Morgan et al., 1989	6	Schweiger et al., 2017	23	Gosner, 1960; Regueira et al., 2015	42	Nieuwkoop and Faber, 1994
30	opercular fold maximum extension	9	Schweiger et al., 2026.; Morgan et al., 1989	9	Schweiger et al., 2017	25	Gosner, 1960; Regueira et al., 2015	45	Nieuwkoop and Faber, 1994
31	first perforation of th opercular fold	13	Schweiger et al., 2026.; Morgan et al., 1989	9.5	Schweiger et al., 2017	41	Gosner, 1960; Regueira et al., 2015	57.5	Nieuwkoop and Faber, 1994
32	both forelimbs are completely errupted	15	Schweiger et al., 2026.; Morgan et al., 1989	10	Schweiger et al., 2017	42	Gosner, 1960; Regueira et al., 2015	58	Nieuwkoop and Faber, 1994
VI	optic suite								
33	optic bulges	5	Schweiger et al., 2026.; Morgan et al., 1989	4.5	Schweiger et al., 2017	not available	-	22	Nieuwkoop and Faber, 1994
34	pupil visible	7	Schweiger et al., 2026.; Morgan et al., 1989	6	Schweiger et al., 2017	not available	-	27	Nieuwkoop and Faber, 1994
35	dark eye pigment	7	Schweiger et al., 2026.; Morgan et al., 1989	6	Schweiger et al., 2017	not available	-	33	Nieuwkoop and Faber, 1994
36	choroid fissure closed	9	Schweiger et al., 2026.; Morgan et al., 1989	9	Schweiger et al., 2017	not available	-	40	Nieuwkoop and Faber, 1994
37	eye lids present	22	Schweiger et al., 2026.; Morgan et al., 1989	14	Schweiger et al., 2017	not available	-	-	Nieuwkoop and Faber, 1994
VII	adult mouth suite								
38	mouth slit before eye	10	Schweiger et al., 2026.; Morgan et al., 1989	8.0	Schweiger et al., 2017	42	Gosner, 1960; Regueira et al., 2015	62	Nieuwkoop and Faber, 1994
39	mouth slit extends to the mid of the eye	14	Schweiger et al., 2026.; Morgan et al., 1989	9	Schweiger et al., 2017	44	Gosner, 1960; Regueira et al., 2015	63	Nieuwkoop and Faber, 1994
40	mouth slit behind the eye	18	Schweiger et al., 2026.; Morgan et al., 1989	12	Schweiger et al., 2017	45	Gosner, 1960; Regueira et al., 2015	64	Nieuwkoop and Faber, 1994
VIII	thyroid gland suite								
41	thyroid anlage present	not available	-	8	Naumann et al., 2021	not available	-	-	
42	follicle present	10	this study	10	Naumann et al., 2021	not available	-	-	
43	follicular wall cells present	10	this study	10	Naumann et al., 2021	not available	-	-	
44	follicular wall cells increse in thickness	12	this study	10	Naumann et al., 2021	not available	-	-	
45	follicular wall cells maximum thickness	13	this study	10	Naumann et al., 2021	not available	-	-	
46	colloid present	12	this study	11	Naumann et al., 2021	not available	-	-	
IX	others								
47	hatching	9.5	Schweiger et al., 2026.; Morgan et al., 1989	15	Schweiger et al., 2017	20	Gosner, 1960	35	Nieuwkoop and Faber, 1994

## Results

### Development of the thyroid gland

For the histological description and measurements, sections of one specimen each for developmental stage 10, 12, 13, 15, 17, 19, 21 and 22 were analysed (Figs. 5 and 6).

**Fig. 2:**
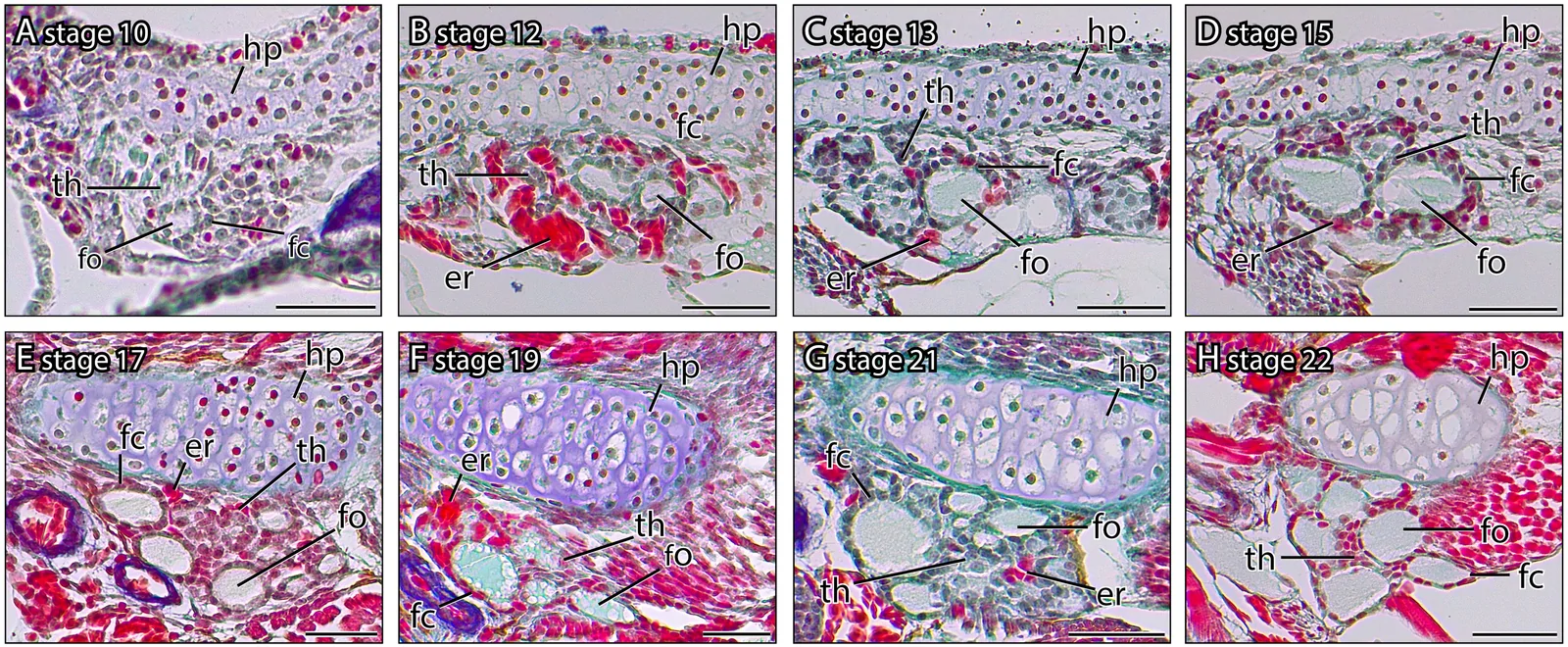
Histological cross sections of the ventral head region of *Arthroleptella villiersi* at different developmental stages showing thyroid gland development. Scale bars equal approximately 400 μm. er, erythrocytes; hp, hyobranchial plate; fc, follicle cell; fo, follicle; th, thyroid gland

The thyroid gland of *A. villiersi* is first recognizable at stage 10 as a spherical cell condensation ventral to the right and left lateral margin of the developing hyoid plate (Fig. 2A). Follicles, showing only a loosely organized outline by follicular wall cells, are present in histological sections of the specimen at this stage. At stage 12, follicular wall cells are organized as a compact epithelium exhibiting cuboid cell shapes and large spherical nuclei. The presence of a high number of erythrocytes indicates an increased vascularization of the thyroid gland. Faintly stained colloid is first detectable in follicular lumina (Fig. 2B). At stage 13, follicular wall cells are organized in a columnar pattern. Their nuclei exhibit a range of shapes from spherical to ovoid, indicating increased mitotic activity. Staining of colloid inside the follicular lumen is more intense compared to the previous developmental stage (Fig. 2C). At stage 15, histology of the thyroid gland appears similar to the previous developmental stage (Fig. 2D). At stage 17, follicular wall cells exhibit larger amounts of cytoplasm compared to previous developmental stages (Fig. 2E). From this stage on, the histological organization is similar compared to stage 19, stage 21 and stage 22 and resembles the mature configuration (Fig. 2F-H). The number of detectable erythrocytes decreases from stage 17 to stage 21, indicating a decreasing degree of vascularization and thyroid gland activity.

**Fig. 3:**
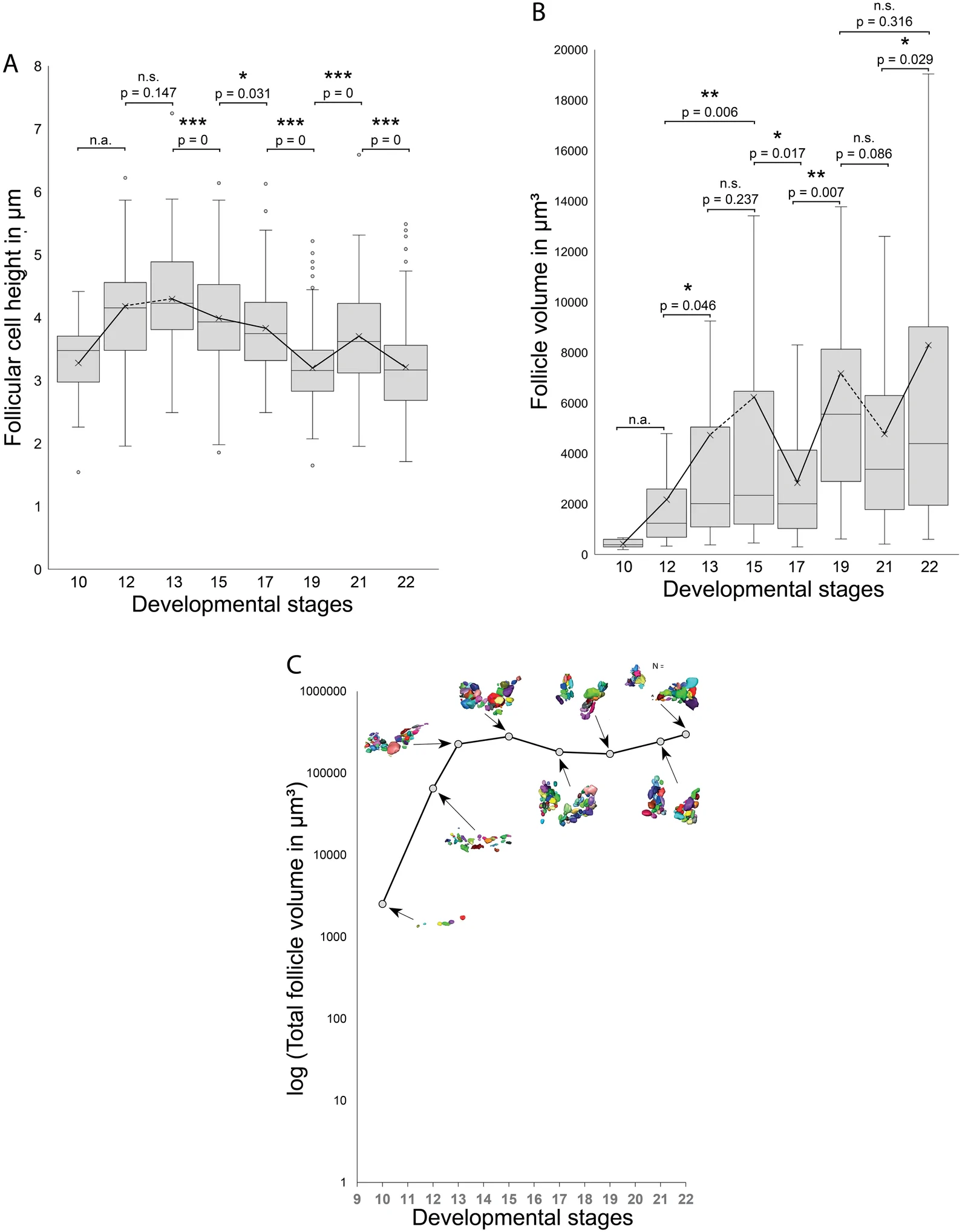
A, Box plot of follicular wall cell heights at different developmental stages. Outliers are shown as dots. B, Box plot of single follicles volumes at different developmental stages. Outliers are not shown due to size constrains but given in Supplementary Material 1. Average values are connected via a solid (significant difference) or dashed line (insignificant difference) in both plots. Non-significant differences are marked with “n.s.”. C, Diagram of total follicle volumes at each developmental stage. The y-axis is logarithmic. Three-dimensional reconstructions of the follicles are shown for each data point. The follicles are depicted in multiple colors to retrace single follicles

Follicular wall cell height, show a distinct pattern (Fig. 3A). The average cell height increases from stage 10 to 12, peaks at stage 13 and then slowly decreases until stage 19. It increases at stage 21 and decreases again at stage 22. The two-sample T-Test shows significant differences of the average follicular wall cell height between stage 10 and 12, 13 and 15, 15 and 17, 17 and 19, 19 and 21, and 21 and 22. Single follicle volumes, increased from stage 10 to 15, decreased from stage 15 to stage 17, again increased again from stage 17 to 19, decreased from stage 19 to 21, and then increased again from stage 21 to 22 (Fig. 3B). The pattern shows an increase from stage 10 to stage 15, followed by a slight decrease to stage 19 and a subsequent increase to stage 22. For measurements and p-values of the two-sample T-Testing see Supplementary Material 1.

### Development of the melanophore pattern

#### Stage 5 to 6 (Fig. 4A)

Only a very few scattered melanophores are detectable in the skin of the dorsal head and trunk at this developmental stage.

#### Stage 7 (Fig. 4B)

Melanophores are present all over the skin of the head and trunk. Most of these melanophores exhibit a stellate morphology with a few short extensions and a broad and flattened central region. Their density is highest at the dorsal midline and decreases ventrolaterally until the level of the eye on the head and the dorsal end of the pronephric system in the trunk. Ventral to that level, melanophores are concentrated in the skin lateroventrally to the eye and overlie the pronephric system. From that level on, melanophore density decreases again ventrolaterally. No melanophores can be seen in the tail skin.

#### Stage 8 (Fig. 4C)

In the trunk, melanophores are present down to a more ventral level compared to the previous developmental stage. A very few of them reach the ventral midline of the trunk. The anterior half of the tail also exhibits melanophores. Their concentration is highest dorsally, decreasing ventrolaterally.

#### Stage 9 (Fig. 4D)

Melanophore density has increased tremendously compared to the previous developmental stage and a higher number of them reaches the ventral midline of the trunk. Tail pigmentation has extended to the anterior two thirds to three quarters of its length.

#### Stages 10 to 14 (Figs. 4E - F)

Density of melanophores increases during this developmental period. In all these stages pigmentation of the tail skin only reaches up the anterior three quarters of the tail length. The posterior quarter of the tail shows only a very few separated melanophores and appears almost unpigmented.

#### Stages 15 to 22 (Figs. 4G – I)

Density of melanophores increases further, reaching a pattern comparable to stage 20-juveniles at around stage 15. Pigmentation of the tail fades in parallel with its degeneration.

**Fig. 4:**
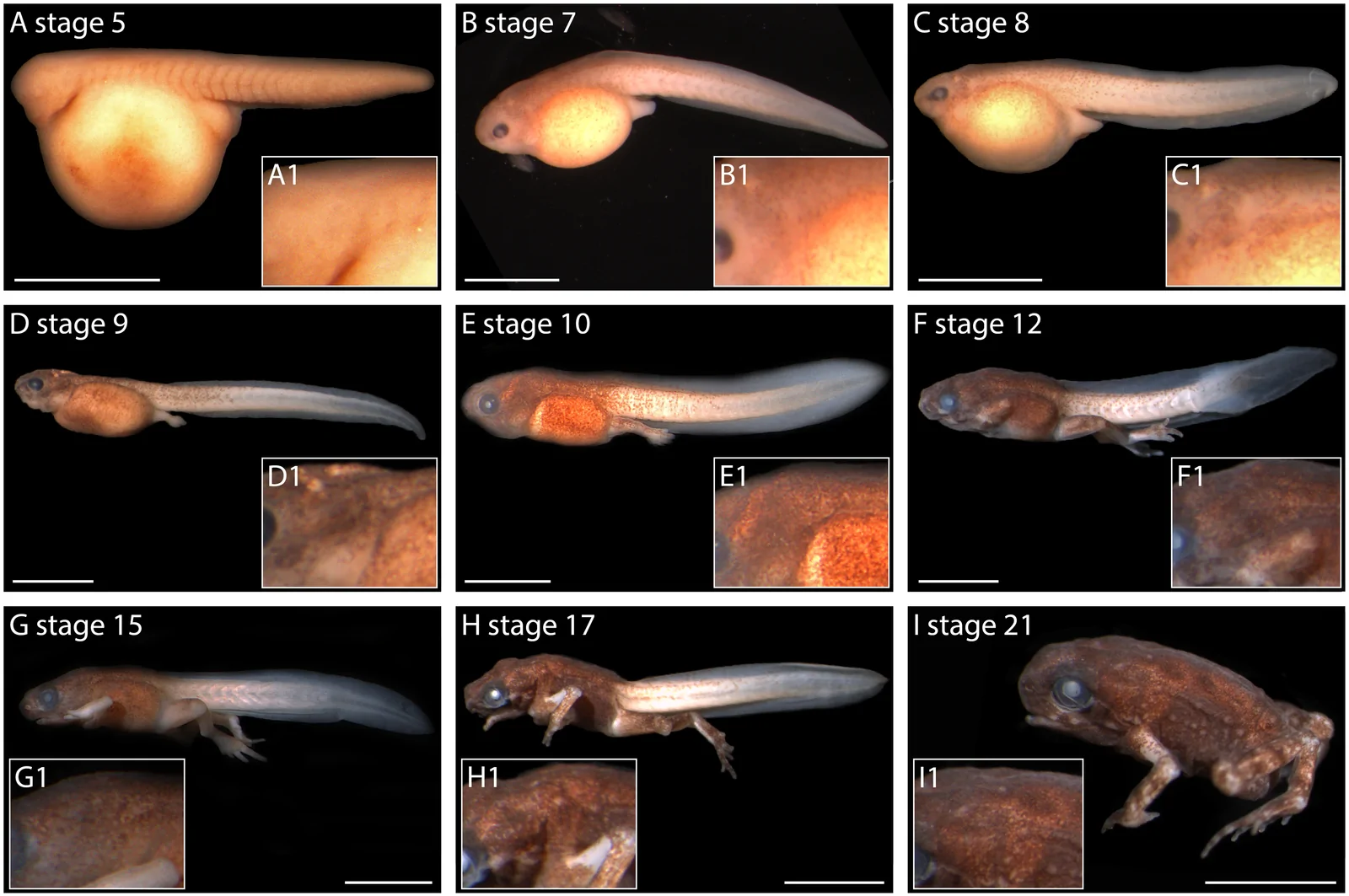
A-I, Photographs of different developmental stages of *Arthroleptella villiersi* in lateral position to illustrate the developing pigmentation pattern. A1- I1, show a close up of the posterior head region of each main photograph. Scale bars equal 2 mm

### Developmental histology of the skin

**Fig. 5:**
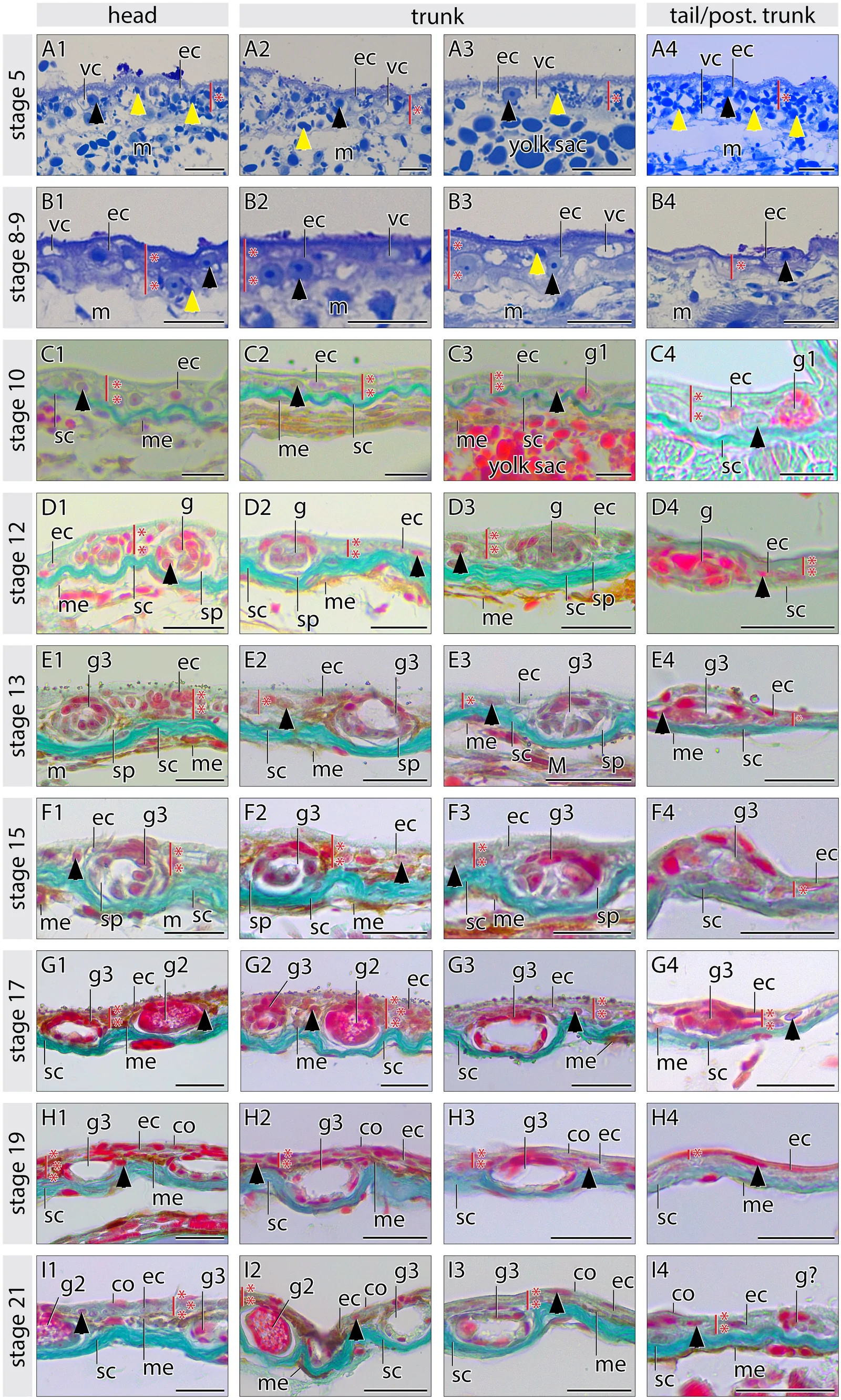
**A-I**, Histological cross sections of the skin of *Arthroleptella villiersi* at different developmental stages. Yellow arrows indicate intracellular yolk droplets. Black arrows indicate epidermal nuclei. The number of epidermal cellular layers is indicated by a red line and asterisks. Scale bars in histological sections equal approximately 200 μm. co, Stratum corneum; ec, epithel cell; g, multicellular gland progenitor; g1, unicellular gland; g2, multicellular granular gland; g3, multicellular mucus gland; g?, degenerating gland; m, mesenchyme; me, melanophore; sc, Stratum compactum; sp, Stratum spongiosum; vc, vacuole

**Stage 5 to 6 (Fig.** 5**A): Head** – The epidermis is single-layered with a sometimes indistinct border between the underlying cells. Epidermal cells are large and cuboid with spherical nuclei, large vacuoles and a high number of intracellular yolk droplets. **Trunk** – Dorsally, the epidermis is mostly single-layered with a few patch-like two-layered areas. Epidermal cells of head and trunk are similar. Ventrally, the epidermis is single-layered. The cells are more flattened compared to the dorsal trunk region. They exhibit large spherical nuclei, large vacuoles, intracellular yolk droplets and are clearly separated from the underlying yolk sac. **Tail** – The epidermis is single-layered and cells are similar compared to the trunk.

**Stage 8 to 9 (Fig.** 5**B): Head** – Dorsally, the epidermis is two-layered. Ventrally, most of the areas are two-layered except for a few single-layered patches. The epidermis is separated from the underlying cells by a sharp border. However, a stratum compactum cannot be detected in this developmental phase. The cells are still large and cuboid with spherical nuclei. The number of vacuoles and intracellular yolk droplets is greatly reduced compared to the previous developmental stage. **Trunk** – The epidermis is two-layered. Dorsally, the external cell-layer is more flattened compared to the internal layer of cuboid cells. Medially, the two layers are more or less similar. Ventrally, both cell layers are more flattened, and nuclei show an ovoid shape. The number of vacuoles and intracellular yolk droplets is also reduced. **Tail** – The epidermis is single-layered. The cells are flattened with ovoid-shaped nuclei and almost no vacuoles and intracellular yolk droplets.

**Stage 10 (Fig.** 5**C): Head** – Dorsally, the epidermis is two-layered and a dense area of melanophores underlies a thin stratum compactum (internal melanophores). Ventrally, a few patch-like single-layered areas are still present overlying a thin stratum compactum. The number of melanophores decreases towards the ventral midline. No vacuoles and only very few scattered intracellular yolk droplets are present. **Trunk** – The epidermis is mostly two-layered. Epidermal cells are more flattened compared to previous developmental stages. Most nuclei exhibit an ovoid-shape. The stratum compactum is distinct. Neither vacuoles nor intracellular yolk droplets can be found dorsally. Laterally, their number increases, resulting in a high number of intracellular yolk droplet in the ventral epidermis. Melanophores underlying the stratum compactum are only present dorsally. **Tail** – The epidermis is single- to two-layered. Cells in the external layer are flattened with ovoid nuclei, while cells in the internal layer are more cuboid with round nuclei. At the tail tip, cells become cuboidal. A stratum compactum is present. A few scattered gland cells are detectable in the middle area and close to the tail tip. No melanophores are recognizable.

**Stage 12 (Fig.** 5**D): Head** – The epidermis is two-layered. The stratum compactum has increased in thickness. Anlagen of large multicellular glands, recognizable as large round cell clusters often sunken into the stratum compactum, are present in the dorsal area. Epidermal organization as a stratum spongiosum is first detected around multicellular gland complexes. **Trunk** – Dorsally, the epidermis is single- to two-layered. Laterally, the epidermis is single-layered with a decreasing density of melanophores. Laterally, most areas of the epidermis are single-layered. Ventrally, the epidermis is single-layered. A dense layer of melanophores underlies the stratum compactum and a stratum spongiosum is first recognizable. **Tail** – Cells in both epidermal layers appear more flattened compared to previous developmental stages. Anlagen of multicellular glands can be recognized. The epidermis at the posterior-most tail area and the tip is single layered.

**Stage 13 (Fig.** 5**E): Head** – The epidermis is single- to two-layered with more areas being single-layered compared to the previous developmental stage. In the two-layered areas, cells of the internal cell layer have a more cuboid shape and nuclei appear more rounded indicating an increased mitotic activity. The number of multicellular glands has increased and some of them can be identified as mucus glands showing a cellular wall surrounding a large central lumen. **Trunk** – The epidermis is single- to two-layered. In the two-layered regions, cells of the internal layer appear cuboid in shape, with round nuclei similar to the head region. Cells of the external layer are more flattened, and their nuclei are more ovoid in shape. The number of multicellular glands has increased in the dorsal and lateral skin and their differentiation appears more pronounced compared to the head. **Tail** – The epidermis is single- to two-layered consisting of flat cells. The number of developing multicellular glands has increased. However, they appear much less differentiated compared to the multicellular glands present in the epidermis of the trunk and head.

**Stage 15 (Fig.** 5**F): Head** – The number of cell layers and the shape of epidermal cells is similar compared to the previous stage. The stratum compactum appears thicker, and the number of multicellular glands has increased compared to the previous stage. Most of them can be clearly identified as mucus glands. **Trunk** – As for the head, the number of cell layers and cellular shapes are similar to the previous stage, but the stratum compactum appears thicker compared to previous developmental stages. The number and stage of differentiation of mucus glands has increased compared to the previous developmental stages. **Tail** – The epidermis appears similar to the previous stage except that the number of multicellular glands has decreased.

#### Stage 17 (Fig. 5G)

From this developmental stage onwards, the skin of the embryo very much resembles the skin of juvenile frogs at stage 20. **Head** – Number and size of mucus glands have increased, and first multicellular granular glands are present in the dorsal epidermis. Additionally, a dense layer of melanophores is present in the dorsal epidermis, lying externally to the stratum compactum (external melanophores). **Trunk** – Dorsally, most areas of the epidermis are three-layered, thinning to a two- or single-layered epidermis towards the ventral midline. Similar to the epidermis of the head, the number and size of multicellular mucus glands have increased, the first multicellular granular glands are present in the dorsal region, and a dense layer of external melanophores has formed, decreasing in a ventral direction. **Tail** – The epidermis appears mostly similar to the previous stage. The number of multicellular glands has further decreased.

**Stage 19 (Fig.** 5**H): Head** – In some patchy areas of the dorsal and lateral sides of the head, the dorsal epidermis is three-layered. The external cell layer is flattened and represents the stratum corneum. A slightly higher number of multicellular mucus and granular glands is present compared to previous stages. **Trunk** – The skin is similar compared to the previous stage except for a stratum corneum, a higher number of multicellular mucus and granular glands and an increased thickness of the stratum compactum. **Tail** – The skin is similar compared to the previous developmental stage.

**Stage 21 (Fig.** 5**I): Head** – The epidermis is mostly similar to the previous developmental stage. The density of external melanophores has increased in the dorsal epidermis, while there are still none in the ventral epidermis. The number of granular glands has increased compared to previous developmental stages. **Trunk** – The epidermis is similar to the previous developmental stage except for a higher density of outer melanophores and an increased number of multicellular granular glands. **Posterior trunk** – The tail has been completely resorbed. The epidermis overlaying the posterior trunk region is two-layered, exhibiting only inner melanophores.

### Neuromast development

**Fig. 6:**
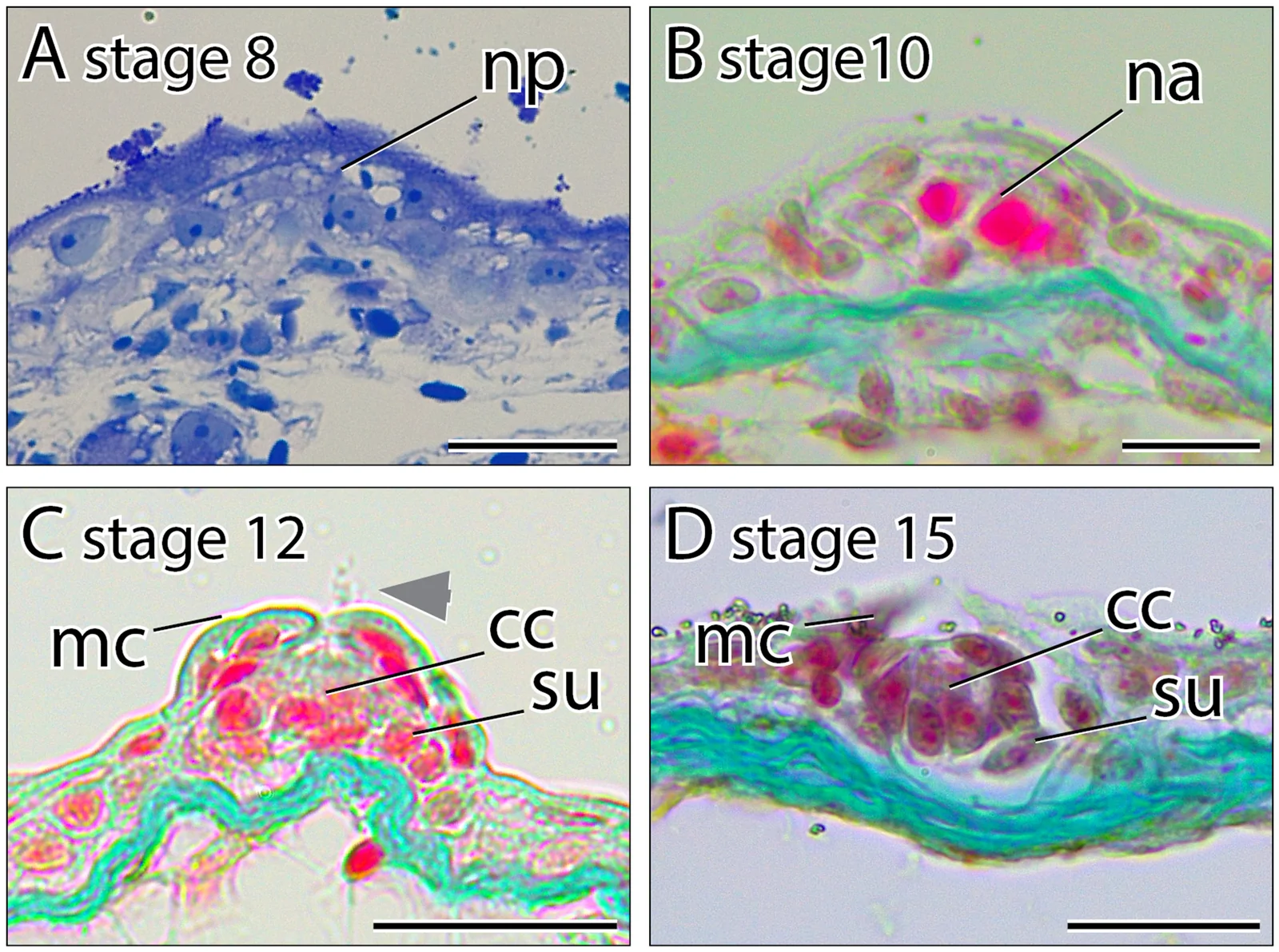
**A-D**, Histological cross sections of the skin of *Arthroleptella villiersi* at different developmental stages to show the development of neuromasts. The grey arrow in C indicates the neuromast cilia. Scale bars in histological sections equal approximately 200 μm. cc, cilium cell; mc, mantle cell; np, neuromast placode; su, support cell

At stage 8, there are a few patches of densely clustered cells visible that protrude slightly from the rest of the dorsal trunk epidermis. We interpret them as early neuromast placodes (see Fig. 6A). At stage 10, the neuromast anlagen can be clearly recognised by their rosette-like shape, and they are present in positions similar to the placodes in the previous stage (Fig. 6B). At stage 12, mature neuromasts are present in the positions of the anlagen described in stage 10. Ciliated median cells are surrounded by non-ciliated support cells and outer-most mantle cells in a rosette-like pattern. The mantle cells protrude centrally to form an externally opening cavity enclosing the support and ciliated cells (Fig. 6C). At stage 13, neuromasts are still present. However, most of them have lost their cilia, indicating degenerative processes comparable to those of the epidermal cells of the tail tip (Fig. 6D). Neuromasts are lacking at stage 15 onwards.

### Heterochrony analysis of skin development in anurans with different developmental modes

**Fig. 7:**
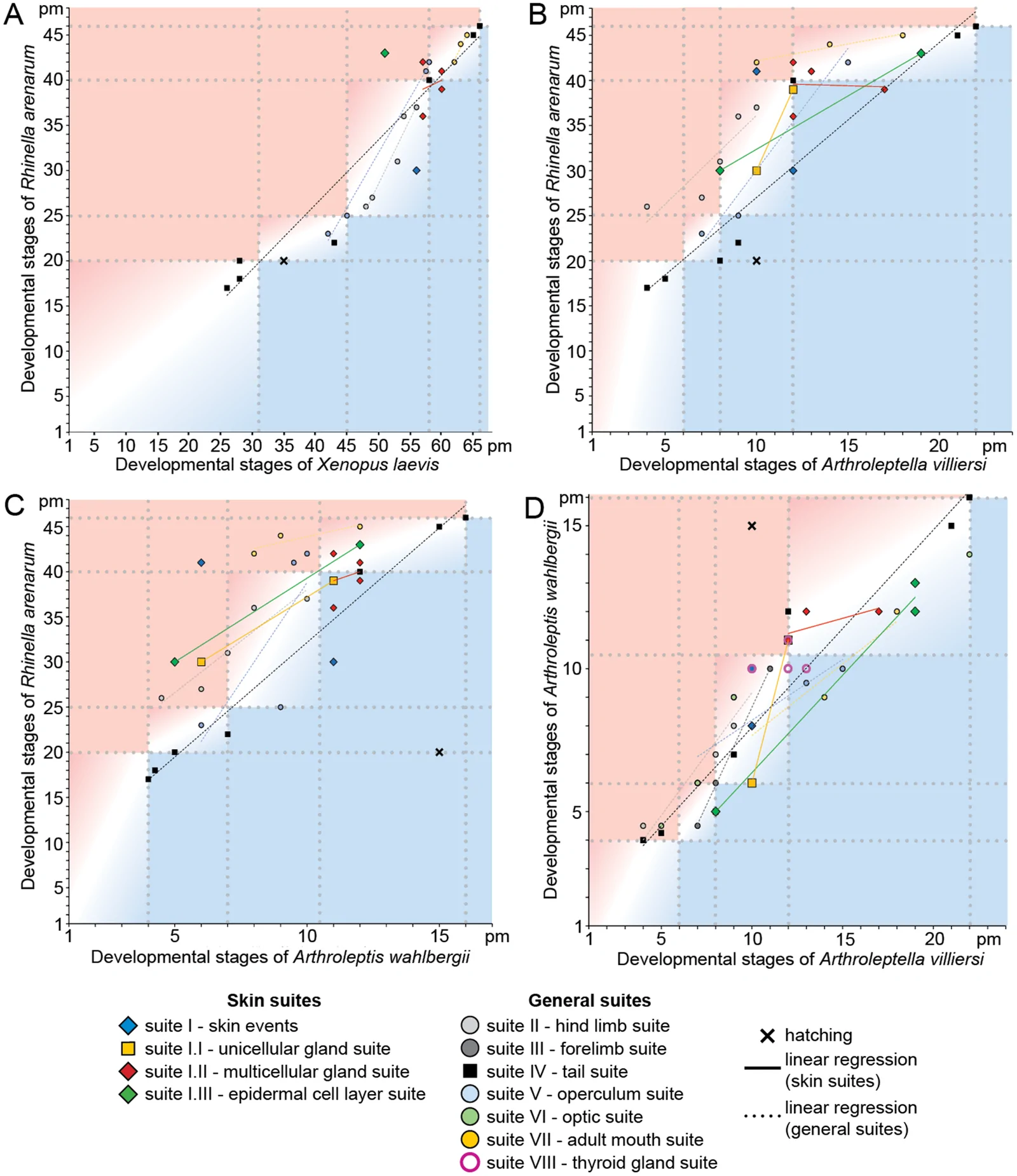
Heterochrony plots of anuran species with different reproductive modes. Light red background in the upper left area of a plot indicates pre-displacement/early development of an event, light blue background in the lower right area of a plot indicates post-displacement/later development of an event in the species on the x-axis. The white area indicates a comparable developmental timing. pm, post-metamorphic

In addition to the plots of the indirect-developing *Xenopus laevis* and *Rhinella arenarum* (Fig. 7A) we tested whether heterochronic shifts correlate with terrestrial reproductive modes. Data from the terrestrial indirect-developing *A. villiersi* were plotted against *R. arenarum* (Fig. 7B). An additional plot was prepared for the terrestrial direct-developing *A. wahlbergii* against *R. arenarum* to test if heterochrony patterns of terrestrial indirect-developing *Arthroleptella* and direct-developing *Arthroleptis* are similar compared to the ancestral condition (Fig. 7C). An additional heterochrony plot of *A. villiersi* against *A. wahlbergii* was prepared to test the similarity of both terrestrial reproductive modes (Fig. 7D). In this case neither of the species represents an ancestral state compared to the other. This heterochrony plot only shows similarities and differences between the species without any phylogenetic connection. Therefore, the terms “earlier” and “later” instead of “pre-displaced” and “post-displaced” (as in the other two plots) are used to avoid the implication that *Arthroleptis* evolved from a terrestrial-indirect developing ancestor that was similar to *A. villiersi*. A summary of the heterochronic shifts detected in the plots is given in Table 3. The timing of developmental events within each suite and events defining the ontogenetic phases are given in Tables 1 and 2.

**Table 2:** General developmental events that are used as criteria to define homologous ontogenetic phases compared in the heterochrony plots

#-Plot	ontogenetic phases	criteria (start of phase)	*Rhinella arenarum* (Gosner-stage, 1960)	*Xenopus laevis* (Nieuwkoop and Faber-stage, 1994)	*Arthroleptella villiersi* (Schweiger-stage, 2026)	*Arthroleptis wahlbergii* (Schweiger-stage, 2017)
a	pre-pharyngula (early embryonic)	fertilized egg	1	1	0	1
b	pharyngula and post-pharyngula (late embryonic)	optic bulge; gill arches present; tailbud elongated	20	31	6	4
c	“tadpole” (pre- and prometamorphosis)	pupil visible, iris dark/black; maximum extension of the opercular fold; foot paddle-shaped	25	45	8	7
d	metamorphosis	begin of teil regression	40	58	12	10.5
e	postmetamorphosis	tail completely resorbed	46	66	22	16

**Table 3:** Summary of heterochronic shifts detected in the heterochrony plots

event/suite number	developmental events	timing in *X. laevis* compared to *R. arenarum*	timing in *A. villiersi* compared to *R. arenarum*	timing in *A. wahlbergii* compared to *R. arenarum*	timing in *A. villiersi* compared to *A. wahlbergii*
I	skin suite				
1	stratum compactum starts to develop	-	-	-	earlier
2	stratum compactum shows increased thickness	pre-displaced	-	post-displaced	earlier
3	intracellular yolk granules absent	-	-	-	-
4	dermal melanophores present	-	pre-displaced	pre-displaced	later
I.I	unicellular gland cell suite				
5	unicellular mucus glands present	-	-	pre-displaced	later
6	unicellualr mucus glands absent	-	-	post-displaced	-
I.II	multicellular gland cell suite				
7	stratum spongiosum present	pre-displaced	-	-	-
8	multicellular gland anlagen	-	-	post-displaced	-
9	multicellular granular glands present	post-displaced	post-displaced	post-displaced	-
10	multicellular mucus glands present	-	-	-	-
I.III	epidermal cell layer suite				
11	epidermis has up to two layers	-	-	-	-
12	epidermis has three or more layers	-	-	-	-
13	stratum corneum present	pre-displaced	-	pre-displaced	-
II	hind limb suite				
14	fist anlage of the hind limb	-	pre-displaced*	pre-displaced	earlier
15	starting elongation of the hind limb buds	-	pre-displaced	pre-displaced	-
16	limb paddle present	-	-	-	-
17	toe 3-5 distinct	-	-	-	-
18	toe 1-5 present	-	-	-	-
III	forelimb suite				
19	first anlage of the forelimb buds	-	-	-	earlier
20	starting elongation of the forelimb buds	-	-	-	-
21	all 4 fingers differentiated	-	-	-	-
IV	tail suite				
22	tail bud present	-	-	-	-
23	starting elongation of the tail bud	-	-	post-displaced	earlier
24	fin folds present	-	-	-	-
25	vascularized translucent fin folds	-	post-displaced	-	-
26	tail begins to regress	-	-	-	-
27	tail regressed to small stump	-	-	-	-
28	tail resorbed	-	-	-	-
V	opercular fold suite				
29	opercular fold first evident	-	-	-	-
30	opercular fold maximum extension	-	-	-	-
31	first perforation of the opercular fold	-	-	pre-displaced	later
32	both forelimbs are completely errupted	-	-	pre-displaced	later
VI	optic suite				
33	optic bulges	-	-	-	earlier
34	pupil visible	-	-	-	-
35	dark eye pigment	-	-	-	-
36	choroid fissure closed	-	-	-	-
37	eye lids present	-	-	-	-
VII	adult mouth suite				
38	mouth slit before eye	-	pre-displaced	pre-displaced	-
39	mouth slit extends to the mid of the eye	-	-	pre-displaced	later
40	mouth slit behind the eye	-	-	-	-
VIII	thyroid gland suite				
41	thyroid anlage present	-	-	-	-
42	follicle present	-	-	-	-
43	follicular wall cells present	-	-	-	-
44	follicular wall cells increse in thickness	-	-	-	-
45	follicular wall cells maximum thickness	-	-	-	later
46	colloid present	-	-	-	-
IX	others				
47	hatching	-	post-displaced	post-displaced	earlier

## Discussion

### Qualitative and quantitative data indicate a conserved pattern of thyroid gland maturation in *A. villiersi*

Metamorphosis in anurans and amphibians generally, is initiated through the activation of the BPT-axis, with TH as an important messenger [34]. In accordance with a previous work on *Arthroleptella bicolor* Hewitt, 1926 [35], we could detect the histological appearance of the thyroid gland at stage 10 and persistent follicular colloid from stage 12 onward. Vascularization increased at stage 12, declining from stage 17. This sequence is comparable to the patterns in both aquatic-indirect and the terrestrial direct-developing species [11, 36].

To obtain a more detailed image of thyroid gland activity during the development of *A. villiersi*, we collected quantitative histological data. Follicular wall cell height, regulated by pituitary secreted thyroid-stimulating hormone, is often used as an indicator for TH synthesis, with higher cells indicating higher synthetic activity [27]. In some amphibians, cell height increases at the onset of metamorphosis, then decreases before the metamorphic climax when high concentrations of TH, stored inside the follicles, are released into the vascular system [11, 26, 37, 38]. This matches our finding in *A. villiersi*, where cell height increases from stage 10 to stage 13 (before metamorphic skin rearrangement) and decreases from stage 13 to 19, correlating with the establishment of adult skin histology. The decrease beginning at stage 13 aligns with a slight delay in single follicle volume reduction from stage 15 to 17, possibly reflecting colloid mass-release into the vascular system and partial follicle collapse.

However, as only one individual was examined per stage, stability of these patterns remains to be tested. While the pattern is intriguing, there are also studies on aquatic indirect-developing species that have found no direct correlation between follicular cell height and TH synthesis and release [36, 39–41]. Overall, follicular cell height and volume offer only coarse indicators of thyroid activity. More robust conclusions require integrating these metrics with qualitative traits such as intra-follicular colloid, the degree of vascularization, expression of TH-receptor beta, and TH-concentration measurements in the blood [42].

### Tadpole-specific skin characters and their metamorphic rearrangement are conserved in terrestrially developing anurans and correlate with thyroid gland maturation

While the anuran skin harbours various chromatophore types, our study focuses on melanophores only because they are the most accessible type using histology. In aquatic indirect-developing anurans, a tadpole-specific melanophore pattern develops first, followed by metamorphic changes in location and type to produce the adult pattern [43, 44]. A similar but more condensed melanophore rearrangement also occurs in the terrestrial direct-developing *Arthroleptis* [11].

In *A. villiersi*, melanophore patterning can be divided into two phases as described for other anurans [11, 43]. The first phase, characterized by an increase in pigmentation before thyroid differentiation, resembles the establishment of the tadpole-specific coloration pattern. The second phase, characterized by skin darkening that correlates with thyroid maturation, parallels the metamorphic shift to the adult pattern. This indicates, that both phases are conserved in terrestrially developing species. We found no embryonic epidermal melanophores or morphologically distinct melanophore populations described in other taxa [11]. Tadpole-like skin development correlates with hatching (stage 8/9; stage 3 in Morgan et al. [18]). At stage 12, metamorphic rearrangement begins, marked by unicellular gland degeneration, multicellular gland complex differentiation, and epidermal thinning to a single layer. Proximal tail skin also shows signs of metamorphic rearrangements (developing multicellular glands and a stratum corneum) despite tail degeneration starting from stage 17 and indicating TH competence. Postmetamorphic characters, like a three-layered epidermis, thickened stratum compactum, stratum spongiosum, stratum corneum, higher melanophore density, and multicellular mucus and granular glands, develop from stage 13 to 19.

**Fig. 8:**
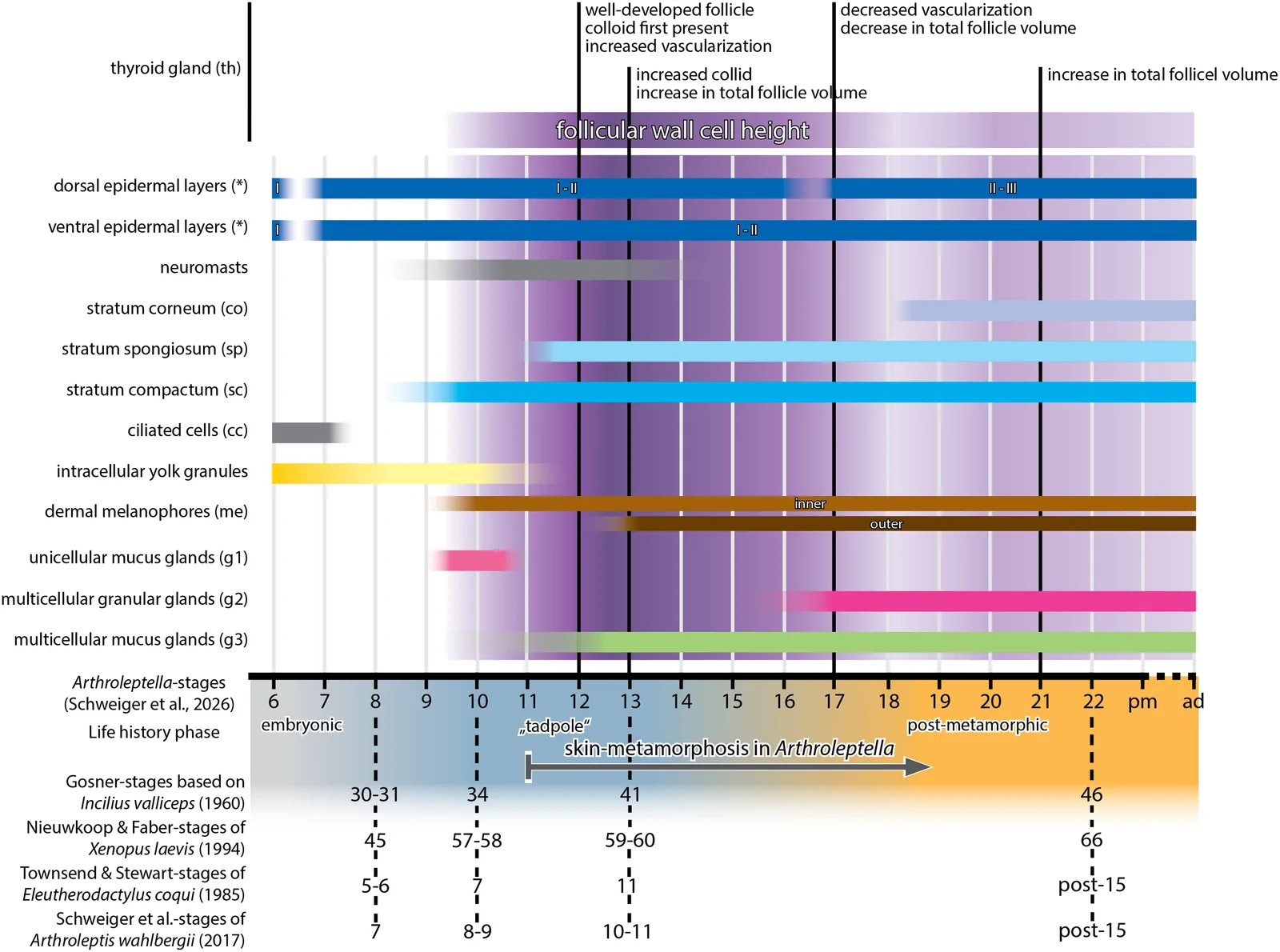
Schematic summary of the skin composition of *Arthroleptella villiersi* at different developmental stages. The histologically estimated thyroid gland activity based on follicle cell height is indicated in different shades of purple. White to light purple indicates a lower activity while dark purple indicates higher activity. The timing of major events of histological differentiation of the thyroid gland are indicated with black lines. To facilitate comparison with other anurans, general developmental stages according to Gosner ([33]; *Incilius valliceps*), Nieuwkoop & Faber ([31]; *Xenopus laevis*), Townsend and Stewart ([12]; *Eleutherodactylus coqui*) and Schweiger et al. ([14]; *Arthroleptis wahlbergii*) are provided as reference points for the presence of paddle-shaped feet (Gosner-stage 30–31), the presence of all fingers and toes mit minimal indentitions between them (Gosner-stage 34), the emergence of both arms from the operculum (Gosner-stage 41) and the end of metamorphosis (Gosner-stage 46). ad, adult; pm, post-metamorphic juvenile

In summary, thyroid maturation correlates with overall skin development and metamorphic rearrangement in *A. villiersi*, consistent with patterns in *A. lightfooti* and some aquatic indirect- and terrestrial direct-developing taxa [11, 18, 21, 32, 45–48]. The data from *A. villiersi* confirm the descriptions for *A. lightfooti* by Morgan et al. [18] and add information on the exact timing of several developmental events, including intracellular yolk granule resorption, dermal melanophore patterning, unicellular and multicellular gland complex differentiation (Fig. 8).This answers the first question posed in this study – skin development and metamorphic rearrangement is largely conserved in *A. villiersi* compared to aquatic indirect-developing species. Additionally, its correlation with thyroid gland maturation indicates a conserved dependency on TH. However, only functional inhibitor and activator assays would be able to confirm a causal link between thyroid gland maturation and metamorphic skin rearrangement.

### Terrestrialization of development precedes neuromast loss in anurans

Neuromasts have been reported in *Arthroleptella* species, but without detailed developmental data [17, 18]. In *A. villiersi*, neuromast placodes appear at stage 8, differentiate until stage 12 (histologically resembling functioning neuromasts of other anuran species e.g. *Pseudis paradoxa* Linnaeus, 1758 [49]), begin to degenerate at stage 13, and are absent at stage 15. Degeneration correlates with histological thyroid maturation, a pattern shared with aquatic indirect-developing species [50]. It differs substantially from the terrestrial direct-developing *Arthroleptis* and *Eleutherodactylus* in which neuromasts never develop [11, 12, 51].

We propose two explanations for the conserved developmental pattern we observed in *A. villiersi*: 1) neuromasts aid hatchlings in detecting movements within the jelly nest fluid, or 2) they are vestigial and possibly non-functional, representing an intermediate stage towards complete loss. The latter could be tested by examining neuromast-associated neural components of the central and peripheral nervous system of *A. villiersi*. The brief presence of neuromasts in *A. villiersi* and their absence in direct-developers supports the view that the lateral line system constitutes a distinct developmental module [51].

### Heterochrony and the evolution of skin development in anurans

By comparing the timing of key metamorphic events (hindlimb development, degeneration of the pronephros, skin metamorphosis, forelimb emergence, tail development and degeneration) with exogenous feeding, hatching and BPT-axis maturation in *A. lightfooti*,* Xenopus* and *Eleutherodactylus*, Morgan et al. [18] concluded that *A. lightfooti* displays a developmental pattern temporally intermediate between direct-developing *Eleutherodactylus* and aquatic indirect-developing *Xenopus*. However, their study lacked skin developmental data for *Eleutherodactylus* or other direct developers. In our present study we used heterochrony plots as a formalized method to test Morgan et al.’s interpretation with the now available detailed skin developmental data of *A. villiersi* and the direct-developing species *A. wahlbergii*.

Formalized heterochrony analyses served as valuable tools for describing ontogenetic shifts and identifying coevolving developmental modules in anurans [52–55]. Methods such as event-pairing [52, 56, 57], heterochrony plots [4], and sequence-heterochrony graphs [52] enable such comparisons. Event-pairing is particularly suitable for broad phylogenetic analyses that include several taxa and ancestral state reconstructions [58]. Heterochrony plots in contrast, allow direct comparison of two taxa and provide clear visualization of covariant characters and identify potential units of evolution. Given the limited skin data in anurans and our focus on developmental modules and units of evolution, we adopted the latter approach.

This also offers the possibility for a broad scale comparison of developmental events between two taxa where a direct stage-by-stage comparison is not possible. The unique developmental mode of *Arthroleptella* differs substantially from the aquatic, indirect development of species underlying the Gosner system ([33]; based on the bufonid *Incilius valliceps*), from the Nieuwkoop & Faber system [31] of *Xenopus laevis*, and from the terrestrial direct-developing models of Townsend & Stewart [12] based on *Eleutherodactylus coqui* and of Schweiger et al. [14] based on *Arthroleptis wahlbergii*. Direct, stage-by-stage comparisons between *Arthroleptella* and these taxa would, for many stages, be largely restricted to hind limb morphology. Such comparisons therefore risk oversimplifying development, as they do not capture the pronounced temporal mosaic of characters typical of direct-developing frogs. Indeed, as shown by Morgan et al. [18], comparisons of *Arthroleptella bicolor* with the Gosner system focus primarily on hind limb development and provide limited insight into other developmental traits. For these reasons, we did not attempt to equate stages across the different staging systems of Gosner-, Nieuwkoop & Faber-, Townsend & Stewart- and Schweiger et al.-stages. In addition to the gross comparison of developmental events facilitated by the heterochrony plots comparable “land mark” stages are given in Fig. 8.

Our results generally support the view that the development of *Arthroleptella* represents an “intermediate” stage between aquatic indirect and terrestrial direct development. Distinct (telescoping) heterochronic shifts include: [1] earlier dermal melanophore appearance in terrestrial reproducing species, with *Arthroleptis* first; [2] unicellular mucus glands developing similarly in indirect-developers but appearing earlier and persisting longer in direct-developing *Arthroleptis*; [3] post-displacement of multicellular granular gland differentiation in terrestrial reproducing species; and [4] pre-displacement of stratum corneum development in *Arthroleptis* compared to the indirect-developing *R. arenarum* and *A. villiersi*. These shifts suggest that anuran skin comprises multiple developmental modules. The neuromast/lateral line system represents one such module that is typically lost in direct-developing taxa [11, 12, 51], retained in some aquatic adults [31, 59], or modified (e.g., in the position and number of neuromasts) in some taxa [60] without altering overall skin organization. Additional modules likely include dermal melanophores, unicellular glands, and at least two multicellular gland types (Table 3).

This helps to answer the second question on the pattern of heterochronic shifts of skin development in anurans with different reproductive modes. Notably, the number and magnitude of heterochronic shifts are greater in the direct-developing *Arthroleptis* than in the terrestrial indirect-developing *A. villiersi* when both are compared to the aquatic indirect-developing condition represented by *R. arenarum*. This pattern mirrors previous analyses of other anurans, which similarly reported a higher degree of heterochronic rearrangement of external characters in direct-developing anurans than in taxa retaining a free-living tadpole phase [24]. The comparatively low number of shifts detected in *A. villiersi* is therefore consistent with its retention of a tadpole-like ontogenetic phase and an overt metamorphosis, whereas direct development is associated with a more extensive temporal reorganization of developmental events.

At the same time, the observed shifts are not randomly distributed. In both *A. villiersi* and *Arthroleptis*, the onset of metamorphic skin remodelling correlates closely with thyroid gland maturation, suggesting that the developmental timing of skin differentiation remains linked to the activity of the BPT-axis [11]. The possible involvement of TH in skin metamorphosis has been suggested in TH-inhibition experiments carried out in the direct-developing *Eleutherodactyls coqui* but has not been investigated in detail [16]. We here show that thyroid gland maturation (evaluated based on maximum follicular wall cell height) appears earlier in *Arthroleptis* compared to *A. villiersi*. The correspondingly earlier appearance of several adult skin characters in *Arthroleptis* may therefore reflect a TH-dependency of skin metamorphosis. This interpretation is consistent with the hypothesis that TH-dependent developmental processes represent integrated developmental modules whose evolutionary trajectories are constrained or channelled by shared endocrine regulation. Consequently, we hypothesize that individual skin characters can exhibit limited heterochronic shifts and their overall evolutionary pattern remains coordinated through a conserved dependence on TH signalling.

## Conclusion

The present study provides a detailed description of thyroid gland and skin development in the terrestrial indirect-developing anuran *Arthroleptella villiersi.* This anuran exhibits a conserved metamorphic rearrangement of the skin, including neuromasts, that correlates with thyroid gland maturation and shows some minor heterochronic shifts, leading to a pattern intermediate between aquatic indirect- and terrestrial direct-developing anurans. Our findings suggest that the evolutionary shift to terrestrial reproduction in anurans can occur without major reorganization of skin development, including its timing. Nonetheless, broader comparative datasets with detailed temporal resolution across additional taxa *—* such as aquatic indirect-developing species (to test for heterochronic shifts independent from terrestrial reproduction) and terrestrial indirect-developing and direct-developing species (to test whether the patterns reported for *A. villiersi* and *A. wahlbergii* are representative for both developmental modes) *—* are needed.

## Supplementary Information

Below is the link to the electronic supplementary material.

Supplementary Material 1

## Data Availability

All data supporting the conclusions of this article are included within the article and its additional files.
